# Depression among inmates in a regional prison of eastern Nepal: a cross-sectional study

**DOI:** 10.1186/s12888-017-1514-9

**Published:** 2017-10-23

**Authors:** Gambhir Shrestha, Deepak Kumar Yadav, Nidesh Sapkota, Dharanidhar Baral, Birendra Kumar Yadav, Avaniendra Chakravartty, Paras Kumar Pokharel

**Affiliations:** 1 School of Public Health and Community Medicine, B.P Koirala Institute of Health Sciences, Sunsari, Dharan, Nepal; 2 Department of Psychiatry, B.P Koirala Institute of Health Sciences, Sunsari, Dharan, Nepal

**Keywords:** Depression, Inmates, Regional prison, Suicide

## Abstract

**Background:**

Depression is the most common form of mental disorder among inmates, with a prevalence much higher than in the general population. This study aims to estimate the prevalence of depression among inmates and identify factors associated with it.

**Methods:**

This cross-sectional study was conducted in Jhumka Regional Prison, the largest prison in eastern Nepal, from September 2014 to August 2015. A total of 434 randomly selected inmates were interviewed using a semi-structured questionnaire examining socio-demographic characteristics, detention status, self-reported health problems, substance use status, and suicidal ideation. Depression was screened using the Center for Epidemiologic Studies Depression scale. Chi-square tests and multiple logistic regression analysis were applied to determine the association between depression and related variables.

**Results:**

The mean age of the participants was 35.7 years (SD 13.3). The prevalence of depression among the inmates was 35.3%. Approximately 2.3% reported suicidal ideation during imprisonment and 0.9% had attempted suicide inside the prison. In bivariate analysis, depression was significantly associated with previous incarceration (OR = 1.91, 95% CI = 1.05–3.47, *p* = 0.033), poor self-rated health (OR = 1.75, 95% CI = 1.16–2.64, *p* = 0.007), frequent appointments when encountering health problems (OR = 1.66, 95% CI = 1.06–2.61, *p* = 0.028), suicidal ideation (OR = 4.44, 95%CI = 1.13–17.44, *p* = 0.038) and loss of weight (OR = 1.49, 95% CI = 1.00–2.23, *p* = 0.049). However, only previous incarceration (AOR = 1.97, 95% CI = 1.04–3.74, *p* = 0.037) and frequent appointments when encountering health problems (AOR = 1.61, 95% CI = 1.01–2.57, *p* = 0.046) remained significant in a multivariate model.

**Conclusions:**

This study showed a high rate of depression among inmates in Nepal. The results suggest a need for psychiatric and rehabilitative care in correctional settings to improve the health status of the inmates.

## Background

More than 10.2 million people are held in penal institutions throughout the world (2014). Among these individuals, the United States has the highest prison population approximately 2.24 million [1]. According to the Department of Prison Management, Nepal had 16,315 prisoners as of mid-year 2014, whom 15,152 (92.9%) were males, 1163 (7.1%) were females and 953 (5.8%) were foreigners [2]. Accordingly, the prison population rate was 48 per 100,000 of the national population. The official capacity of the prison system in Nepal is 6416; hence, the occupancy level is 254%. This figure clearly depicts the issue of overcrowding in prisons [2].

The prevalence of mental health problems is higher in the prison population than in the general population [3–5]. The World Health Organization estimated that, of the 9 million prisoners worldwide, at least 1 million (11%) suffer from significant mental disorders, and the most common mental health problems are depression and anxiety [6]. Mental health problems are the most common cause of morbidity in prisons, which create a major challenge for prison management [7]. A systematic review that included 23,000 prisoners from 62 surveys examining serious mental disorders showed that 3.7% of men had psychotic illnesses, 10% had major depression, and 65% had a personality disorder. Among women, 4.0% had psychotic illnesses, 12% had major depression, and 42% had a personality disorder [3].

Prison settings such as overcrowding, lack of privacy, violence, social isolation, inadequate mental health facilities, and the effects of the prison sentence may lead to mental disorders among prisoners during imprisonment [8]. These disorders may be present even before admission to prisons and may be exaggerated by prison settings. Suicidal ideation represents an early expression of vulnerability to self-harming behaviors and suicide [9, 10]. Many studies have shown that suicide is the most common cause of mortality in prisons [11–14], and several studies have identified depression or depressive symptoms as an important risk factor for self-harm [9, 13, 15, 16]. A cross-sectional survey among 996 Australian inmates found that one-third of inmates reported lifetime suicidal ideation and one-fifth had attempted suicide [17].

Prisoners come from the community and will return to the community. Therefore, the prevention and rehabilitation of mental disorders should not be neglected in prison settings. Assessing and addressing the mental health needs of prisoners will aid in the development of appropriate policy and health services to improve the health of prisoners. These measures will further help to reintegrate inmates into community life [8].

This study is the first in Nepal to estimate the prevalence of depression among inmates in prisons and to determine its associated factors.

## Methods

### Study setting and design

This was a cross-sectional study carried out in Jhumka Regional Prison from September 2014 to August 2015. The prison is situated in Jhumka, Sunsari district of the Eastern Development Region of Nepal. It is the largest adult male correctional facility in the Eastern Development Region of Nepal, with a capacity of holding 1500 prisoners (recently upgraded in March 2014 from the previous capacity of 500 prisoners). Among 1203 inmates present in the prison during the study period, 749 were convicted and the rest were under trial.

### Inclusion criteria

Inmates who had spent at least three months in the prison were included in the study, while transferred inmates with a stay of less than three months in the Jhumka prison were excluded from the study. It takes a few months (two to three months) to adjust to prison life, and psychiatric symptoms generally stabilize during this period [18, 19]. This occurrence might be attributed to factors such as safety, structure, a lack of drug and alcohol consumption, and access to medication and healthcare services in the prison [18]. Additionally, these prisoners are usually detained for forensic observation and transferred to another prison [19].

### Sample size

This study is a part of a larger research study to assess the overall health status of the inmates in Jhumka Regional Prison. In a similar study by Nobile et al., the disease with the lowest prevalence, based on self-reported health status, was diabetes (15.1%) [20]. By using the formula for finite population, sample size was calculated as follows;

Sample size = {Z^2^ × PQ/L^2^}/[1 + {(Z^2^ × PQ/L^2^)/ Total no. of inmates}] = 544/(1 + 544/1203) = **375.**

P is the prevalence from reference study, Q is the complement of P, i.e., Q = 100-P, L is allowable error, which is taken to be 20% of P in this study and Z is the standard normal variate, which is 1.96 for 95% confidence interval. The total number of inmates in Jhumka Regional Prison at the time of the study was 1203.

Thus, adding 20% for possible non-response, the final sample size is 450 participants.

### Sampling technique

The total sample size included in this study was 450. A list of all eligible inmates was collected from the prison authority to construct a sampling frame for the study. Simple random sampling was done to select the sample unit using random numbers generated from Microsoft Excel 2007. For selected inmates who were not present at the time of the interview, the sample units were followed by three attempts to include them in the study.

### Methods of data collection

Data were collected by the corresponding author via face-to-face interviews using semi-structured questionnaires examining socio-demographic characteristics, detention status, self-reported health problems, substance use status, prisoners’ perceived health status and suicidal ideation. The covariates were chosen based upon prior studies [20]. Privacy was maintained during the interview.

### Center for Epidemiologic Studies Depression Scale

The validated Nepali Center for Epidemiologic Studies Depression (CES-D) scale was used to screen the depressive status of the inmates. The CES-D is a widely used 20-item scale, designed to measure current levels of depressive symptoms. A score of 16 or greater on the CES-D is the cut-off to indicate major depressive symptomatology [21]. A diagnosis of depression was not made; rather this was a screening for the presence of depressive symptoms. The CES-D scale has been used in other prison studies [22–25].

### Operational definitions

The variables in the study were categorized as per the available literature to provide greater ease of comparison. The socio-demographic characteristics comprised of age (less than 40 years and 40 and above), religion (Hindu and others), residence (urban and rural), marital status (unmarried and ever married), and employment prior to incarceration (unemployed and employed). Level of education was classified according to the Ministry of Education 2010 as illiterate (those who could not read and write), primary education (Grade1–5), secondary education (Grade 6–10) and higher secondary and above (Grade 11 and above) [26]. Economic status of the family was categorized into below the poverty line, which is per capita income of less than 1.25 dollars per day per person, and above the poverty line, with per capita income of greater than or equal to 1.25 dollars per day per person (1 US dollar = 100 Nepalese rupees) [27].

Offense type was classified into the following categories:

1. Violent crime, including murder, female trafficking and kidnapping.
2. Sexual offense, including forced sexual intercourse (rape) encompassing both psychological coercion and physical force, and attempted rape [28].
3. Drug crime, including the use, possession, manufacture, or distribution of drugs classified as having a potential for abuse [28].
4. Property crime, including burglary, motor vehicle theft, or theft. This category includes both attempted and completed crimes [28].
5. Other crimes, including fraud, wildlife and forest crime, and other non-violent crimes.

Detention status was classified into convicted prisoners (prisoners who have been given a sentence for imprisonment for a definite period of time) and under-trial prisoners (prisoners who are imprisoned on remand while awaiting trial in a court of law, i.e., not yet sentenced) [29].

Other imprisonment characteristics were duration of prison stay (less than one year, one to 5 years and more than 5 years), previous incarceration (yes or no) and number of prisoners per cell.

Substance use disorders prior to incarceration, such as alcohol, tobacco, illicit drugs and injectable drugs, were reported as ‘yes’ or ‘no’. Self-rated health status was assessed on a Likert scale consisting of the following options: excellent, very good, good and poor. Later, these responses were dichotomized into good/very good and poor. The participants were asked if they had any current health problems and any health problems at entry. Comparison of present health with health at entry was dichotomized into unchanged/improved and worsened. Arrangement of appointments when encountering health problems was assessed on a Likert scale consisting of the following options: never, rarely, sometimes and often. Later, the never and rarely categories were merged. Loss of weight was assessed by asking the prisoners whether they had lost weight during the last 3 months of incarceration. Suicidal characteristics comprised of whether they had suicidal ideation at any time during imprisonment, had attempted suicide before and/or during imprisonment.

### Statistical analysis

All data were entered in Microsoft Excel 2007 software and analyzed in the Statistical Package for Social Sciences (SPSS, version 17). The dependent variable was significant depressive symptoms, as assessed by the CES-D scale, and the independent variables were sociodemographic characteristics, incarceration profile, substance abuse, suicidal characteristics, health-related problems and access to health care. The internal consistency of the CES-D scale was measured by Cronbach’s alpha, which was found to be 0.91.

Descriptive statistics were used to show the prevalence of depression symptoms and other variables. Chi-square tests and binary logistic regressions were performed to determine the association between depressive symptoms and the independent variables. A *p*-value of <0.05 was considered as the cut-off point for statistical significance. Independent variables significant at *p* < 0.20 were further analyzed with multiple logistic regression to determine the strength of association between the variables.

### Ethical consideration

The study was approved by Institutional Ethical Review Board, B.P Koirala Institute of Health Sciences. Permission to conduct the study in the prison was granted by the Department of Prison Management, Ministry of Home Affairs, Government of Nepal, Kalikasthan, Kathmandu, Nepal. Written approval to conduct the study in the prison was taken from the Jailer of Jhumka Regional Prison. Written informed consent from the participants was received prior to the interview. Prisoners were assured of confidentiality, and they were informed that participation was voluntary and that they could withdraw at any time of the interview without giving reasons for doing so. They were told that their acceptance or refusal to take part in the study would not affect their incarceration or the possibility of parole.

## Results

The total number of inmates in the prison was 1203 at the time of the study. Out of 450 randomly selected inmates, 434 gave consent for the study, with a non-response rate of 3.6%. The age of the participants ranged from 18 to 81 years, with a mean of 35.73 years (SD 13.25). Most of the inmates (88.7%) were the first-time offenders, and the rest had been reincarcerated. Only 15.7% of the inmates were under-trial and the rest had been convicted.

### Depression among inmates

A total of 153 (35.3%) inmates scored at or above the cut-off value for depression. The mean CES-D score was found to be 12.38 (SD 9.03), ranging from 0 to 41. However, only 21 (4.8%) inmates self-reported having any psychological disorders, excluding substance abuse.

### Suicidal characteristics among inmates

Only 10 (2.3%) inmates in this study reported having suicidal ideation during imprisonment, and four of them had attempted suicide in prison. For comparison, seven inmates had also attempted suicide before imprisonment (Table 1).

**Table 1:** Suicidal characteristics among inmates in a regional prison in eastern Nepal

Suicidal characteristics	Categories	Frequency (*n*)	Percentage (%)
Suicidal ideation during imprisonment	Yes	10	2.3
	No	424	97.7
Attempted suicide during imprisonment	Yes	4	0.9
	No	430	99.1
Attempted suicide before imprisonment	Yes	7	1.6
	No	427	98.4

### Depression and socio-demographic characteristics

In bivariate analysis, the rate of depression among the inmates was not found to be associated with socio-demographic characteristics, such as age, religion, marital status, residential area, education, prior occupation and economic status (Table 2).

**Table 2:** Association of depression among inmates with socio-demographic characteristics

Sociodemographic characteristics	Categories	Depression *n* (%)		OR	95% CI	*p*-value
		Present	Absent			
Age in years	< 40	103 (33.8)	202 (66.2)	Ref		0.320
	≥ 40	50 (38.8)	79 (61.2)	1.24	0.81–1.90	
Religion	Hindu	127 (36.0)	226 (64.0)	Ref		0.510
	Others	26 (32.1)	55 (67.9)	0.84	0.50–1.41	
Residence	Urban	57 (30.2)	132 (69.8)	Ref		0.051
	Rural	96 (39.2)	149 (60.8)	1.49	1.00–2.23	
Marital status	Unmarried	47 (34.8)	88 (65.2)	Ref		0.898
	Ever married	106 (35.5)	193 (64.5)	1.03	0.67–1.58	
Education	Illiterate	35 (37.6)	58 (62.4)	Ref		
	Primary	34 (40.5)	50 (59.5)	1.13	0.62–2.06	0.699
	High school	65 (31.7)	140 (68.3)	0.77	0.46–1.28	0.316
	Intermediate and above	19 (36.5)	33 (63.5)	0.95	0.47–1.93	0.896
Employment status	Employed	109 (33.7)	214 (66.3)	Ref		0.262
	Unemployed	44 (39.6)	67 (60.4)	1.29	0.83–2.01	
Economic status	Above poverty line	17 (31.5)	37 (68.5)	Ref		0.535
	Below poverty line	136 (35.8)	244 (64.2)	1.21	0.66–2.24	

### Depression and imprisonment characteristics

There was no association between depression and the type of offense, duration of prison stay and number of prisoners per cell. In this study, inmates with former incarceration were more likely to be depressed than those who were first-time offenders (49.0% vs. 33.5%). This difference was statistically significant (OR = 1.91, 95% CI = 1.05–3.47, *p* = 0.033). There was no association with prevalence of depression between the convicted and under-trial inmates (Table 3).

**Table 3:** Association of depression among inmates by characteristics of imprisonment

Imprisonment characteristics	Categories	Depression *n* (%)		OR	95% CI	*p*-value
		Present	Absent			
Offense type	Drug-related	32 (31.4)	70 (68.6)	Ref		
	Violence	50 (32.3)	105 (67.7)	1.04	0.61–1.78	0.882
	Sexual offense	42 (40.0)	63 (60.0)	1.46	0.82–2.58	0.196
	Property	12 (38.7)	19 (61.3)	1.38	0.60–3.18	0.448
	Others	17 (41.5)	24 (58.5)	1.55	0.73–3.28	0.252
Duration of prison stay in years	≤ 1	33 (31.1)	73 (68.9)	Ref		
	1–5	82 (35.2)	151 (64.8)	1.20	0.74–1.96	0.464
	> 5	38 (40.0)	57 (60.0)	1.48	0.83–2.64	0.190
Previous incarceration	No	129 (33.5)	256 (66.5)	Ref		0.033*
	Yes	24 (49.0)	25 (51.0)	1.91	1.05–3.47	
Detention status	Convicted	131 (35.8)	235 (64.2)	Ref		0.586
	Under-trial	22 (32.4)	46 (67.6)	0.86	0.49–1.49	
No. of prisoners per cell	≤ 50	111 (36.9)	190 (63.1)	Ref		
	> 50	42 (31.6)	91 (68.4)	0.79	0.51–1.22	0.287

*significant at *p* < 0.05

### Depression and substance use disorder prior to incarceration

The prevalence of depression in this study was not significantly associated with substance misuse prior to incarceration (Table 4).

**Table 4:** Association of depression among inmates with substance use status

Substance use	Categories	Depression *n* (%)		OR	95% CI	*p*-value
		Present	Absent			
Alcohol	No	39 (32.8)	80 (67.2)	Ref		
	Yes	114 (36.2)	201 (63.8)	1.16	0.74–1.82	0.506
Smoking status	No	46 (36.5)	80 (63.5)	Ref		
	Yes	107 (34.7)	201 (65.3)	0.93	0.60–1.43	0.726
Tobacco chewing	No	53 (34.6)	100 (65.4)	Ref		
	Yes	100 (35.6)	181 (64.4)	1.04	0.69–1.58	0.844
Illicit drug user	No	118 (37.5)	197 (62.5)	Ref		
	Yes	35 (29.4)	84 (70.6)	0.70	0.44–1.10	0.117
Injectable drug user	No	138 (35.8)	247 (64.2)	Ref		
	Yes	15 (30.6)	34 (69.4)	0.79	0.42–1.50	0.470

### Depression and health status

In this study, depression was more prevalent among inmates who rated their health as poor than those who rated their health as good (40.4% vs. 27.9%), and this difference was statistically significant (OR = 1.75, 95% CI = 1.16–2.64, *p* = 0.007). There was no significant association between depression and current health problems, health problems at entry, and comparison of current health with health at entry. Inmates who reported that they frequently encountered health personnel when they had health problems were more likely to be depressed than those who reported sometimes for this variable (OR = 1.66, 95%CI = 1.06–2.61, *p* = 0.028). The prevalence of depression was found to be higher among inmates with suicidal ideation (70.0%) than those who did not have suicidal ideation (34.4%), and this difference was found to be statistically significant (OR = 4.44, 95% CI = 1.13–17.44, *p* = 0.038).

In this study, 39.3% inmates who reported weight loss during imprisonment were depressed, whereas 30.3% inmates who reported no weight loss were found to be depressed. This relationship was statistically significant (OR = 1.49, 95%CI = 1.00–2.23, *p* = 0.049) (Table 5).

**Table 5:** Association of depression among inmates with health status

Health status	Categories	Depression *n* (%)		OR	95% CI	*p*-value
		Present	Absent			
Self-rated health	Good/ Very good	50 (27.9)	129 (72.1)	Ref		
	Poor	103 (40.4)	152 (59.6)	1.75	1.16–2.64	0.007*
Comparision with health at entry	Unchanged/ Improved	36 (28.8)	89 (71.2)	Ref		
	Worsened	117 (37.9)	192 (62.1)	1.51	0.96–2.36	0.073
Health problems at entry	No	116 (35.7)	209 (64.3)	Ref		
	Yes	37 (33.9)	72 (66.1)	0.93	0.59–1.46	0.741
Current health problems	No	18 (26.1)	51 (73.9)	Ref		
	Yes	135 (37.0)	230 (63.0)	1.66	0.93–2.96	0.082
Arrange appointments when health problem	Sometimes	98 (31.6)	212 (68.4)	Ref		
	Often	46 (43.4)	60 (56.6)	1.66	1.06–2.61	0.028*
	Never / rarely	9 (50.0)	9 (50.0)	2.16	0.83–5.62	0.113
Suicidal ideation in prison	No	146 (34.4)	278 (65.6)	Ref		
	Yes	7 (70.0)	3 (30.0)	4.44	1.13–17.44	0.038*
Loss of weight in prison	No	59 (30.3)	136 (69.7)	Ref		
	Yes	94 (39.3)	145 (60.7)	1.49	1.00–2.23	0.049*

*significant at *p* < 0.05

### Depression and related variables

In the multiple logistic model, only previous incarceration and frequent encounters with health personnel when inmates had health problems were found to be associated with depression. Formerly incarcerated inmates were nearly twice as likely to be depressed than first-time offenders (AOR = 1.97, 95% CI = 1.04–3.74, *p* = 0.037). Similarly, inmates who reported that they often encountered health personnel when they had health problems were more likely to be depressed than those who reported appointments occasionally (AOR = 1.61, 95%CI = 1.01–2.57, *p* = 0.046) (Table 6).

**Table 6:** Multiple logistic regression examining depression among inmates and related variables

Variables	Adjusted odds ratio (AOR)	95% CI	*p*-value
Residence			
Urban	Reference		
Rural	1.27	0.82–1.99	0.286
Previous incarceration			
No	Reference		
Yes	1.97	1.04–3.74	0.037*
Duration of prison stay			
≤ 1 year	Reference		
1–5 years	1.09	0.65–1.84	0.743
> 5 years	1.30	0.70–2.44	0.409
Illicit drug use			
No	Reference		
Yes	0.86	0.51–1.45	0.569
Self-rated health			
Good/Very good	Reference		
Poor	1.42	0.87–2.30	0.160
Comparision with health at entry			
Unchanged / Improved	Reference		
Worsened	1.17	0.68–2.00	0.566
Arrange appointment when health problems			
Sometimes	Reference		
Often	1.61	1.01–2.57	0.046*
Never/ Rarely	2.40	0.88–6.57	0.088
Current health problems			
No	Reference		
Yes	1.24	0.67–2.30	0.486
Suicidal ideation			
No	Reference		
Yes	3.31	0.80–13.64	0.098
Weight loss			
No	Reference		
Yes	1.14	0.72–1.80	0.582

*significant at *p* < 0.05

## Discussion

This is the first study in Nepal, to the best of our knowledge, to investigate the prevalence of depression and its associated factors among inmates in the largest prison in eastern Nepal. This study revealed that 35.3% of the inmates had symptoms of depression similar to those found in studies done in Ukraine, the United States, Nigeria and Iran [30–33]. However, in some studies conducted in Brazil and India, the prevalence of depression was quite low (12% and 18%, respectively) [34, 35]. In a study done by Lekka et al., a high prevalence (75%) of depression was found [36]. These differences can be explained partly by the use of different instruments for assessing depression, and partly by the conditions of the study settings, such as privacy, laws, and cultures. The prevalence of depression is much higher in the prison population compared with the general population in Nepal (4.2%) [37]. In another study done in Dhulikhel, Nepal, the prevalence rate was found to be only 17.3% among the male population, using the same CES-D tool [38].

This study observed a large discrepancy between the rates of self- reported psychological disorders being treated (4.8%) and the high prevalence of depression. This finding is analogous to those of other studies [34, 39], which may be explained by the fact that the majority of health services across the country are devoid of a mental health facility and that mental illness is often stigmatized [40]. In addition, lack in ability among prisoners to recognize their own illness, particularly mental disorders, and thus failing to seek psychiatric treatment, plays a major role.

Among 434 inmates, 4 (0.9%) had attempted suicide during imprisonment, a finding that was similar to those of studies conducted in Canada and Switzerland [41, 42]. However, suicidal ideation and suicidal attempts inside the prison were found to be lower compared to those found in studies done in Australia (9.1% and 2.5%) and Iran(44.6% and 38.9%) [17, 43]. This finding is likely due to high vigilance and daily head counts by the authorities, and less access to any means of committing suicide. Another reason may be that one-third of prison suicides occurs within the first week of custody, which we have excluded from this study [44].

There was no association of depression with the type of offense and the duration of imprisonment, which is consistent with the results found by Fotaye et al. [45]. The reason might be that there was no separation of inmates or difference in behaviours by the correctional authorities according to the type of offense in this prison. We did observe an increase in the prevalence of depression with time spent in prison. With increasing duration of incarceration, the inmates had more physical complaints and mental health problems. On the other hand, with an increasing period of incarceration, there is better adjustment to the prison environment and thus less depression, which may have led to the insignificant association between duration of imprisonment and depression. This conclusion is supported by other studies in which the inmates did not become more depressed, but their mental states improved over time [46–48].

Formerly incarcerated inmates were more likely to have depression. This relationship persisted even after adjusting for other related variables in the multiple logistic regression. The direction of this relationship could not be ascertained within the study’s cross-sectional design. This finding may be partly due to the unavailability of mental health and rehabilitation services in the prison and partly due to the lack of co-ordination in the community to provide appropriate health care services to returning prisoners with depression [49]. The other reason may be that prisoners had to bear the breakdown of relationships and social isolation after being released. This experience may result in further exacerbation of their depressive symptoms and involvement in criminal activities [50].

This study also revealed that inmates who rated their health as poor had a higher prevalence of depression, a finding similar to results from a study conducted in Norway [48]. Depression was likely in prisoners who had current health problems. In contrast, Baumann et al. found that there was no association between depression and self-consideration as ill [22]. It is evident that perceived poor health is associated with poorer mental health.

This study also showed that the inmates who had frequent appointments when they had health problems were more likely to be depressed. This relationship persisted even after adjusting for other related variables in multiple logistic regression. This finding may be explained by the fact that most of the inmates in this study were of low socioeconomic status and may have had difficulty in accessing health services, so the prison may have provided them with the opportunity to access health care [51–54].

Depression was found to be associated with suicidal ideation, which was consistent with findings from other studies [9, 17]. The reasons for this association may be separation from the family, the guilt of the crime, violence in the prison, and an inability to cope with the prison environment, which leads to depression, hopelessness and suicidal ideation [9]. Depression is the strongest predictor of suicide. Therefore, suicidal ideation in individuals suffering from depression should be examined carefully, and adequate mental health services should be provided in prisons [9, 11, 13, 15].

There are some limitations in this study. The participants recruited were from one male prison located in the Eastern Development Region of Nepal; thus, these findings cannot be generalized to other prison populations or to the national prison population. However, this prison is one of the largest regional prisons in Nepal and is comprised of a diverse population from all over the country. Additionally, this study screened for the presence of depressive symptoms rather than major depressive episode diagnoses.

Furthermore, this study does not allow us to make definitive inferences about the effect of the risk factors associated with depression, as it has a cross-sectional design, and psychiatric screening was not performed upon admission to rule out the presence of mental health problems prior to imprisonment.

## Conclusions

The present findings show a high prevalence of depression among the inmates in Jhumka Eastern Regional Prison. This study has also demonstrated a high rate of substance abuse among the inmates. The association of depression with re-incarceration and frequency of health consultation indicates the need for further research and the development of strategies to decrease the rate of re-incarceration. Urgent attention is required to address the mental health problems of the inmates through proper diagnosis and management, along with the use of rehabilitation programs. Steps should be taken to reintegrate prisoners into the community, to ensure the continuation of psychiatric care after release, and to decrease social isolation. These actions may further reduce recidivism and protect individuals, families and the community.

## Abbreviations

AOR: Adjusted odds ratio
CES-D: Center for Epidemiologic Studies Depression
CI: Confidence interval
SD: Standard deviation
